# Active surveillance for highly resistant microorganisms in patients with prolonged hospitalization

**DOI:** 10.1186/s13756-019-0670-8

**Published:** 2020-01-07

**Authors:** Guido J. H. Bastiaens, Tom Baarslag, Corinne Pelgrum, Ellen M. Mascini

**Affiliations:** 1 Laboratory for Medical Microbiology and Medical Immunology, Rijnstate Hospital, Pres. Kennedylaan 100, 6883 AZ Velp, The Netherlands; 2 Department of Medical Microbiology & Radboud Center for Infectious Diseases, Radboud University Medical Center, Nijmegen, the Netherlands; 3 Department of Infection Control, Rijnstate Hospital, Wagnerlaan 55, 6815 AD Arnhem, The Netherlands

**Keywords:** Infection control, Surveillance, Nosocomial, Antimicrobial resistance, Cross infection

## Abstract

We evaluated a new hospital policy comprising active surveillance for highly resistant microorganisms (HRMO) in patients with prolonged hospitalization, including detection of nosocomial transmission after identification of HRMO carriers. Our findings raise the question of whether active surveillance should be extended from traditional risk groups to patients with prolonged hospitalization.

## Introduction

The rising threat of antimicrobial resistance has been recognized worldwide. In the Netherlands, rates of highly resistant microorganisms (HRMO) have traditionally been low [1]. However, even here, antimicrobial resistance is emerging and the Dutch Ministry of Health, Welfare and Sport regards this matter as having the utmost priority [2].

Besides antimicrobial stewardship, hand hygiene, and transmission-based precautions, active surveillance is important to prevent cross-contamination, allowing detection of patients colonized with HRMO, including methicillin-resistant *Staphylococcus aureus* (MRSA), vancomycin-resistant *Enterococcus faecium* (VRE), and carbapenemase-producing *Enterobacteriales* (CPE). Active surveillance for HRMO in patients at risk due to prior admission to foreign hospitals, residence in refugee and migrant centres or exposure to occupational livestock has been endorsed by Dutch guidelines for years [3, 4]. Nevertheless, we are often confronted with patients without risk factors, who are unexpectedly tested HRMO-positive, sometimes even as part of an outbreak [1].

Among other factors, length of in-hospital stay has been positively associated with HRMO-carriage [5, 6]. To identify HRMO-carriage in patients with prolonged hospitalization a new policy comprising active surveillance for HRMO in patients who have been hospitalized ≥14 days has been endorsed. By isolating newly identified, hospitalized HRMO-carriers and by performing source and contact tracing investigations, the new policy aims to reduce transmission of previously unnoticed HRMO in our hospital. We assessed the impact of this new policy.

## Methods

### Hospital setting and study design

This retrospective, nonrandomized observational study was conducted at Rijnstate, an 809-bed, teaching hospital. According to national guidelines [3, 4], patients at risk for HRMO are routinely screened at admission to reduce nosocomial spread. In addition, new policy postulated hospital-wide active surveillance testing for MRSA, VRE and CPE in patients with prolonged hospitalization (≥14 days) and subsequent isolation upon detection. No informed consent was required by the local medical ethics committee as the screening for HRMO at day 14 was part of the new policy and considered as evaluation of care. We analysed data gathered between 15 December 2016 and 15 March 2018. Day 14 of hospitalization was chosen as an optimum in the balance between the number of patients tested versus diagnostic costs.

### Specimen collection and microbiological procedures

On day 14 of hospitalization an instruction appeared in the electronic patient record to collect a single set of swabs from nose, throat, and rectum for HRMO-screening. Screening for MRSA was performed with nose, throat and rectal swab samples, while VRE and CPE colonization was tested in rectal swabs only.

Detection of MRSA carriage was performed as described previously [7] and molecular typing of MRSA-isolates by multiple locus variable number of tandem repeat analysis (MLVA) was performed at the RIVM, Bilthoven, The Netherlands. Detection of VRE was done by quantitative reverse-transcriptase polymerase chain reaction (RT-PCR) of *vanA* and *vanB* genes as described previously [8]. Molecular typing of VRE-positive isolates was performed at the University Medical Center Groningen, Groningen, The Netherlands. Carbapenemase resistance genes (*bla*_KPC_, *bla*_OXA-48_, *bla*_NDM_, *bla*_IMP_ and *bla*_VIM_) were detected by RT-PCR as described previously [9].

Source and contact tracing investigations were performed by the infection-control team according to guidelines endorsed by the Dutch Working Party on Infection Prevention [4].

### Data collection and analysis

A query was run in the General Laboratory Information Management System selecting patients hospitalized ≥14 days, who underwent active surveillance for HRMO. Patient data including department, date of sample collection and microbiological result of active surveillance, were collected. Our primary outcome variable was the number of MRSA, VRE and CPE-carriers identified using active surveillance in patients hospitalized ≥14 days. Secondary outcome variables included the number of newly identified MRSA-positive inpatients hospitalized ≥14 days in relation to the total number of newly identified MRSA-positive inpatients, and the number of contact tracings indicating nosocomial transmission. Data were analysed using Excel (v14.7.2, Microsoft Corporation).

Costs of laboratory services for microbiological determinations were calculated.

## Results

**Fig. 1:**
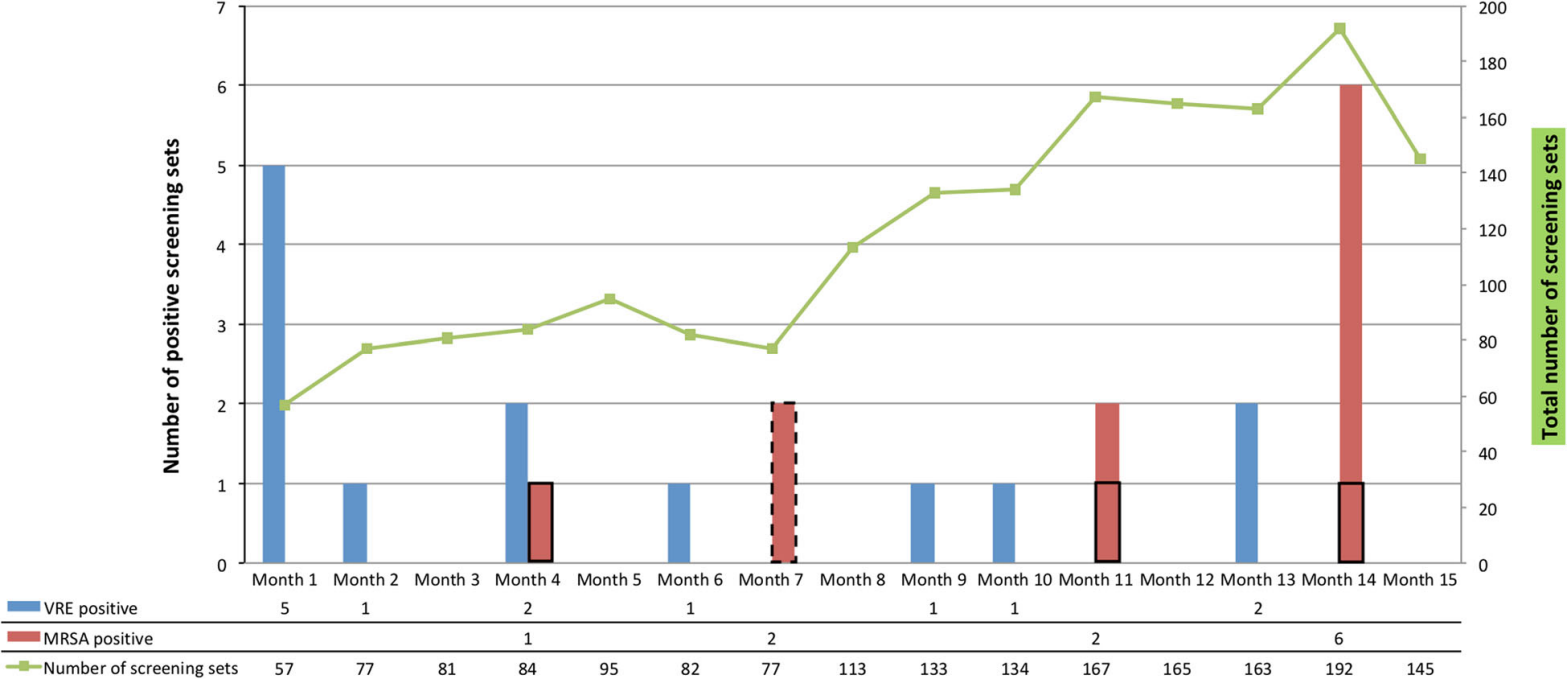
Number of positive screening sets in relation to total number of screening sets in patients hospitalized ≥14 days with active surveillance. Each column represents the number of newly identified positive screening sets and each dot indicates the total number of screening sets per month. The column enclosed by the dotted line represents the MRSA-positive twins from the haematology ward; the columns enclosed by the black lines represent the MRSA-positive patients at the department of geriatrics. Abbreviations: MRSA, methicillin-resistant *Staphylococcus aureus*; VRE vancomycin-resistant *Enterococcus faecium*, VRE

A total of 1899 screening sets from 1765 individual patients were collected and included in our analysis. The number of screening sets sampled each month gradually increased from 60 in the first month after policy change to a peak of 192 in the second last month (Fig. 1). Over the course of 15 months HRMO was detected in 24 previously unidentified patients (1.36% [95% confidence interval {CI}, 0.92–2.02]; Fig. 1).

MRSA colonization was discovered in 11 patients (0.62% [95% CI, 0.35–1.11]). Contact tracing investigations indicated that nosocomial transmission occurred twice. In the neonatology ward a nurse providing care for two twins who were found MRSA-positive in our screening programme (Fig. 1), was tested positive for MRSA as well as both parents. Other healthcare workers screened were MRSA-negative (*n* = 85). Molecular typing confirmed nosocomial transmission showing a similar MLVA-profile in each of the isolates. At the department of geriatrics our surveillance strategy retrospectively identified a cluster of three MRSA-positive patients carrying an identical strain (Fig. 1). Notably, these patients had no overlapping period of hospitalization, suggesting presence of a common source although this was never proven by source and contact tracings including screening of patients and healthcare workers [3].

VRE was detected in 13 previously unidentified patients (0.74% [95% CI, 0.43–1.26]). A monoclonal outbreak of VRE was discovered before our study period and all VRE-isolates were *vanA*-positive belonging to the outbreak strain cgMLST 1026, as confirmed by whole genome sequencing. This is illustrated in Fig. 1 where the number of VRE positive patients is highest in the first month. No CPE-positive patients were detected.

**Fig. 2:**
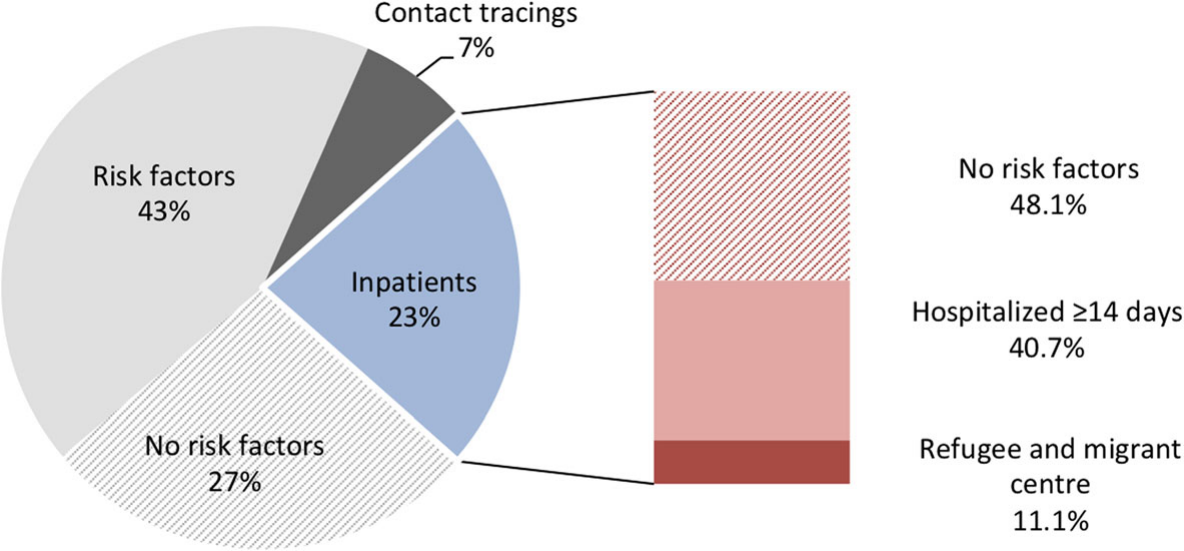
Newly identified MRSA-positive patients. Each slice represents a percentage of a total of 117 MRSA carriers. The grey slices represent outpatients. Each block in the bar represents a percentage of a total of 27 MRSA-positive inpatients

During the study period a total of 117 patients were tested positive for MRSA in our laboratory (Fig. 2). Seventy-seven percent (90/117) of newly identified MRSA carriers were outpatients and 23% (27/117) were hospitalized patients. Among the latter group, active screening of patients hospitalized ≥14 days accounted for 40.7% (11/27) of newly identified MRSA carriers, while 48.1% (13/27) were unexpected results of clinical culture samples and 11.1% (3/27) were MRSA-positive patients from refugee and migrant centres (Fig. 2).

Costs of microbiological laboratory services for active screening of 1000 patients hospitalized ≥14 days were calculated at €120,000, resulting in €120 per screened patient and €227,880 during our study period covering the processing of 1899 screening sets.

## Discussion

Our study shows that active surveillance in patients hospitalized ≥14 days can be used to identify asymptomatic HRMO colonization supporting early detection of nosocomial transmission. Although only 1.4% of screened patients tested positive for HRMO, our surveillance strategy revealed 2 new clusters of MRSA, disclosed 40.7% of newly identified inpatients colonized with MRSA, and detected 13 VRE-positive patients who turned out to be part of a recently recognized outbreak. Due to infection prevention and control measures including enforced hand hygiene and cleaning and disinfection, isolation precautions, clinical lessons to the staff, and microbiological screening of contact patients, further dissemination was prevented.

To prevent nosocomial transmission of HRMO, compliance with hygienic practices including transmission-based precautions and environmental cleaning is of utmost importance. In medical settings with excessive workload where adherence to infection control precautions may be suboptimal, our surveillance programme will have most added value. It supports identification of HRMO carriers, thereby reducing the risk of nosocomial transmission and outbreaks, and it could be used as a proxy for infection control practices.

Our policy brings considerable costs, raising the question whether it is money well spent. Considering high costs of controlling a nosocomial outbreak [10], however, we believe that hospitals may benefit from extending active surveillance from traditional risk groups to patients with prolonged hospitalization as our policy contributes to timely control of HRMO spread. We assume that our program may be optimized by a differentiated approach in detection of HRMO species on selected wards at risk for dissemination of HRMO. Besides, screening of other patient categories that are potentially at risk for HRMO carriage such as patients who are transferred from chronic care facilities may contribute to control of HRMO in hospitals. More detailed, prospective studies are needed to address these issues.

## Data Availability

All data generated or analysed during this study are included in this published article.

## Abbreviations

CPE: Carbapenemase-producing *Enterobacteriales*
HRMO: Highly resistant microorganisms
MLVA: Multiple locus variable number of tandem repeat analysis
MRSA: Methicillin-resistant *Staphylococcus aureus*
RT-PCR: Quantitative reverse-transcriptase polymerase chain reaction
VRE: Vancomycin-resistant *Enterococcus faecium*
